# Identification of a Genome Instability-Associated LncRNA Signature for Prognosis Prediction in Colon Cancer

**DOI:** 10.3389/fgene.2021.679150

**Published:** 2021-06-07

**Authors:** Tengfei Yin, Dongyan Zhao, Shukun Yao

**Affiliations:** ^1^Peking University China-Japan Friendship School of Clinical Medicine, Beijing, China; ^2^Graduate School, Peking Union Medical College and Chinese Academy of Medical Sciences, Beijing, China; ^3^Department of Gastroenterology, China-Japan Friendship Hospital, Beijing, China

**Keywords:** genome instability, long noncoding RNA, colon cancer, prognosis, immune infiltration

## Abstract

Long non-coding RNAs (lncRNAs) were reported to have the potential in maintaining genome instability, but the identification of lncRNAs related to genome instability and their prognostic value have not been largely explored in colon cancer. In this study, we obtained 155 genome instability-associated lncRNAs based on somatic mutation profiles in colon cancer from The Cancer Genome Atlas (TCGA) database. Functional enrichment analysis revealed the possible roles of genes co-expressed with those lncRNAs involved in some cancer, genome instability and immune related biological processes. Combined with overall survival data, a seven-lncRNA signature was established for prognosis prediction. According to the risk score calculated by this signature, high-risk patients characterized by high somatic mutation count, high microsatellite instability, significantly poorer clinical outcomes and specific tumor immune infiltration status compared with low-risk patients. The lncRNA signature was validated to be an independent prognostic indicator with good predictive performance in TCGA cohort. Furthermore, the prognostic value of the ZNF503-AS1 in lncRNA signature was confirmed in another independent dataset from Gene Expression Omnibus database. In summary, the genome instability-associated lncRNA signature in this study could be a promising tool for effectively predicting survival outcomes in colon cancer.

## Introduction

Colon cancer is one of the leading causes of cancer death worldwide (Siegel et al., 2019). The incidence and mortality of colorectal cancer ranked third and second, respectively, according to the global cancer burden report in 2020 (Sung et al., 2021). The early diagnosis rate of colon cancer is relatively low, and more than 50% of patients are diagnosed at an advanced stage, which has posed a greatly increasing threat to human health (Siegel et al., 2020). Although the treatment of colon cancer has revolutionized from surgery, chemotherapy and radiotherapy to immunotherapy and molecular targeted therapy in recent years, these strategies cannot satisfy the ever-rising demand for improving the overall survival (OS) and disease-free survival of patients in light of resistance and relapse. The 5-year survival rate for patients with stage I colorectal cancer can reach 90%, while it is approximately 10% for patients with stage IV disease (Brenner et al., 2014). Classic prognostic indicators such as tumor-node-metastasis (TNM) stage fail to hold ideal efficiency in prognostic prediction in some patients (Nitsche et al., 2011). In clinical applications, it is urgent to discover new effective and reliable prognostic biomarkers to improve the prediction power and accuracy and guide personalized therapy with simplified tools for clinical outcome assessment.

Somatic mutations in the cancer genome, which are cumulative consequences of endogenous and exogenous mutational processes with different strengths from the first division of fertilized eggs into cancer cells, could play a crucial role in cancer initiation, progression and therapeutic resistance (Alexandrov et al., 2013, 2020). With the development of next-generation sequencing technologies, the whole-genome-scale sequencing of bulk tumor samples have increased, including somatic mutation profiling, allowing the discovery of drug targets, prognostic indicators and protocols of target therapies. It was found that 16% of patients with early-onset colorectal cancer had gene mutations (Pearlman et al., 2017). Considering the high frequency and wide spectrum of somatic mutations, it is essential to focus on the crucial functions of somatic mutations in the biological processes of colon cancer. Yang et al. (2019) revealed that recurrent mutations in APC, KRAS, and TP53 made rectal cancer cells resistant to chemoradiotherapy, and preoperative chemoradiotherapy could alter the genome landscape at the somatic mutation and copy number variation levels of rectal cancer. However, the role of somatic mutations in the carcinogenesis of colon cancer has not been largely explored. Microsatellite instability (MSI), caused by a defective mismatch repair system, is characterized by the widespread mutation throughout the genome and particularly clustered in highly repetitive microsatellite region (Dai et al., 2021; Gilson et al., 2021), suggesting that MSI could be used as genome instability index for cancer. Recently, the presence of MSI was demonstrated as a predictive biomarker for immunotherapy and prognosis (Li K. et al., 2020; Zhu et al., 2021). Moreover, tumor-infiltrating immune cells has become one of the important factors affecting clinical benefits of immunotherapy and predicting prognosis of colon cancer (Zhou et al., 2019). But the correlation of genome instability and immune infiltration in colon cancer still needs further analysis.

In recent years, long non-coding RNAs (lncRNAs), a newly identified class of non-coding RNA molecules longer than 200 nucleotides, have been shown to be involved in various regulatory functions in biological processes rather than encoding proteins, and their misregulation could be related to numerous diseases, including cancers (Yang et al., 2014; Chen et al., 2016). An increasing number of studies have revealed that lncRNAs participate in gene activation or silencing through diverse mechanisms, including epigenetic regulation, chromatin modification and genome stability maintenance (Lee et al., 2016; Peng et al., 2017; Wang et al., 2017). Novel lncRNAs have been reported to act as pivotal factors in the promotion or inhibition of carcinogenesis, such as colorectal cancer (Wang et al., 2019; Zhong et al., 2019). Prognostic modeling is a valuable method for cancer management to recognize high-risk patients in a timely manner and avoid unnecessary interventions for low-risk patients. Accumulating evidence has shown that lncRNAs have the potential to become novel biomarkers and prognostic models for the early diagnosis and clinical outcome prediction of colon cancer (Fan and Liu, 2018; Huang et al., 2020; Zhang Y. et al., 2020). Interestingly, Bao et al. (2020) proposed a genomic instability-related lncRNA signature for improving the clinical outcome of breast cancer. However, the relationship between genome instability and lncRNA prognostic modeling has rarely been reported in colon cancer.

The aim of our research was to screen lncRNAs related to genomic instability in colon cancer patients, establish a genomic instability-associated lncRNA signature for predicting clinical outcome, explore the relationship between genome instability and immune cell infiltration, and judge the power of this prognostic model. LncRNAs were utilized as linkages to evaluate genomic instability and predict prognosis in colon cancer. Collectively, we could provide important insight into the mechanism of genomic instability in the tumorigenesis and progression of colon cancer and identify novel biomarker candidates for early diagnosis and clinical outcome predictions in colon cancer.

## Materials and Methods

### Data Collection

The RNA expression profiles, clinical characteristics and somatic mutation data of patients with colon cancer were downloaded from The Cancer Genome Atlas (TCGA) database^1^. A total of 514 RNA expression profile samples were downloaded, including 473 tumor samples and 41 normal samples. Transcriptome data were distinguished according to mRNA and lncRNA profiles. The clinical information of 452 patients with colon cancer was downloaded. After excluding patients with OS less than 30 days and those lacking crucial clinical factors, the information of 418 samples was used for further analysis. The somatic mutation data of 399 patients with colon cancer were downloaded and identified using MuTect software. The MSI score of each colon cancer sample was derived from a previous study (Bonneville et al., 2017). Another independent colon cancer dataset, GSE17538 was reviewed form Gene Expression Omnibus (GEO) database^2^. Platform and series matrix files of GSE17538 were downloaded, then probe matrix was converted into corresponding RNA name using the platform file. LncRNA and mRNA transcript profiles were generated, respectively, based on annotation from the GENCODE database^3^. A total of 229 patients with OS longer than 30 days in GSE17538 were enrolled for further independent validation analysis. This study was approved by the Ethics Committee of China-Japan Friendship Hospital (No. 2018-116-K85-1). An informed consent statement was not necessary because all data were acquired from the TCGA and GEO database and available to the public.

### Screening Genome Instability-Associated LncRNAs

To analyze genome instability, the cumulative mutation frequency of each sample was obtained, as well as the mutation count of each gene in all samples. The top 25% of samples with the highest mutation frequencies were regarded as the high mutation group, that is, the genome unstable (GU) group, and the bottom 25% of samples with the lowest mutation frequencies were regarded as the low mutation group, that is, the genome stable (GS) group. The differentially expressed lncRNAs between the two groups were obtained as genome instability-associated lncRNAs using the Wilcoxon test (false discovery rate (FDR)-adjusted *P* value < 0.05 and | log2 fold change (FC)| > 1). According to the expression levels of the genome instability-related lncRNAs, hierarchical clustering analysis was performed with hclust function in R to classify all samples into two clusters: the genome stable-like (GS-like) cluster and the genome unstable-like (GU-like) cluster. The somatic mutation counts of the two clusters were compared using the Mann-Whitney *U* test. *P* value < 0.05 was the statistical cutoff.

### GO and KEGG Functional Enrichment Analysis

In the co-expression analysis of mRNA profiles and genome instability-related lncRNAs, the Pearson correlation coefficient was calculated, and the top 10 mRNAs most correlated with each lncRNA were selected as target genes. A lncRNA-mRNA co-expression network was visualized. Gene Ontology (GO) and Kyoto Encyclopedia of Genes and Genomes (KEGG) enrichment analyses were performed on mRNAs in the co-expression network using the “clusterProfiler” package in R/Bioconductor, and a *P* value < 0.05 was regarded as a statistical criterion to determine the functions and pathways related to genome instability.

### Establishment of Genome Instability-Associated LncRNAs Signature for Prognosis Prediction

All samples were evenly and randomly divided into two groups, a training set and a testing set, and the differences between the two sets in terms of clinical information, including age, sex and TNM stage, were compared. The expression data of genome instability-associated lncRNAs and OS data were combined, and Cox proportional hazards regression analysis was used to analyze the relationship between lncRNA expression and clinical prognosis. Based on the prognosis-related lncRNAs identified in the training group, a lncRNA signature (lncSig) was obtained as a prognostic model, and then the risk score of each sample was calculated for all patients.

### Correlation Analysis of Genome Instability With Prognosis and Tumor Immune Infiltration in LncRNA Signature

According to the median value of the risk score in the training set as the risk cutoff value, patients with a high risk score were divided into a high-risk group and those with a low risk score were divided into a low-risk group. Next, based on RNA profiles, “CIBERSORT” package was used to quantify 22 immune cells infiltration levels in colon cancer samples with cutoff *P* value < 0.05. Further analyses were performed to explore the relationships between prognostic risk, somatic mutation, MSI, single gene mutations and immune cell infiltration level.

### Efficiency Verification of the LncRNA Signature for Prognostic Stratification

**FIGURE 1:**
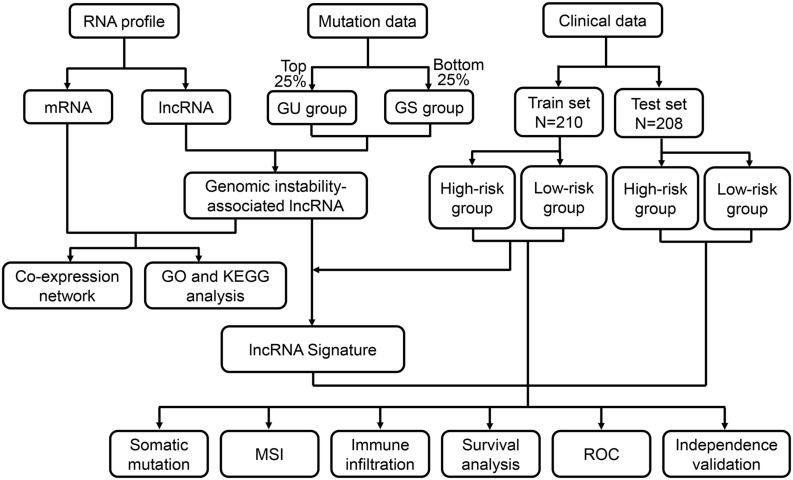
Flow chart of the procedure applied in this study. The RNA profiles, mutation counts and clinical characteristics of colon cancer patients were downloaded from the TCGA database. Genome instability-associated lncRNAs were obtained after the identification of the differentially expressed lncRNAs between the two groups of patients with the top 25% and bottom 25% mutation count frequencies. After identifying the correlated mRNAs, a mRNA-lncRNA co-expression network was constructed, and functional enrichment analysis was performed. After combining the lncRNA data and the overall survival data, a lncRNA signature was developed for clinical outcome prediction. According to the calculated risk score, patients in the training set and testing set were classified into high- and low-risk groups. Correlation of risk score, somatic mutation, microsatellite instability (MSI) and immune infiltration was analyzed. The prognostic prediction efficiency was verified through survival analysis, receiver operating characteristic (ROC) curves and independent validation. GU, genome unstable; GS, genome stable; TCGA, The Cancer Genome Atlas; GO, Gene Ontology; KEGG, Kyoto Encyclopedia of Genes and Genomes.

A series of methods were used for performance analysis and verification of the genome instability-associated lncRNA signature as a prognostic model. First, OS, disease-specific survival (DSS) and progression-free interval (PFI) were evaluated using Kaplan–Meier curves and log-rank test. The cutoff criterion was a *P* value < 0.05. Given that the prognostic model was feasible for all patients, further analyses were conducted to stratify TCGA patients with different clinical statuses and explore the applicability of the model. Time-dependent receiver operating characteristic (ROC) curve analysis was utilized to examine the accuracy of the prognostic model based on the area under the curve (AUC). Next, to evaluate the independence of the prognostic risk score in this model from other clinical factors, univariate and multivariate Cox regression analyses were utilized, and hazard ratios (HRs) and 95% confidence intervals (CIs) were calculated. External validation was also performed using the GSE17538 dataset from the GEO database to explore whether the lncRNAs in lncSig that could be applicable for OS prediction in another independent dataset. The ROC curve was utilized to compare the predictive performance of our signature with other signatures in previous studies. The flow chart of this study is shown in Figure 1.

## Results

### Identification of Genome Instability-Associated LncRNAs

**FIGURE 2:**
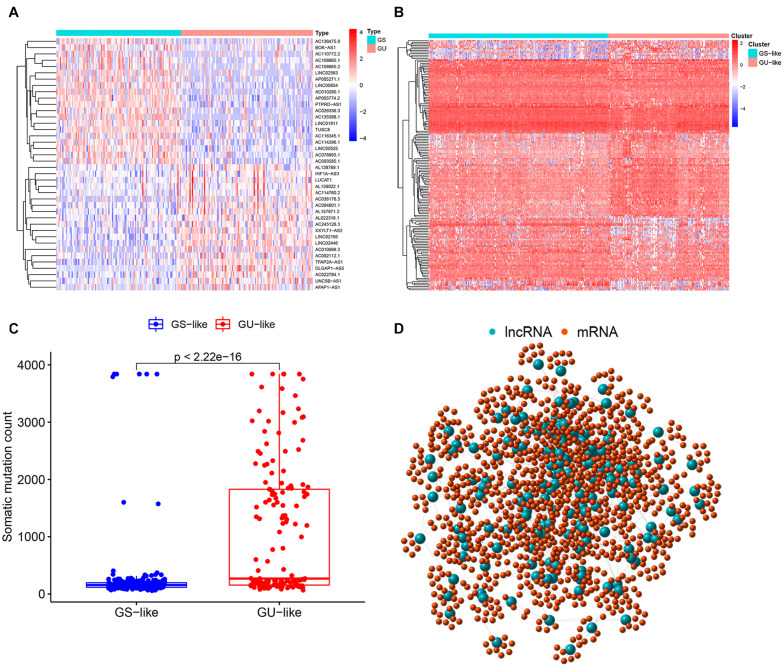
Identification of genome instability-associated lncRNAs and the co-expression network. **(A)** Heatmap of the top 20 genome instability-associated lncRNAs expressing the most upregulation and downregulation. **(B)** Genome stable-like (GS-like) cluster and genome unstable-like (GU-like) cluster of patients arranged by the expression level of genome instability-related lncRNAs. **(C)** Boxplot of somatic mutations of the GS-like cluster and the GU-like cluster. The cumulative mutation count of the GU-like cluster was significantly higher than that of the GS-like cluster. **(D)** Co-expression network of genome instability-associated mRNAs and lncRNAs. The large blue circles represent lncRNAs, and the small red circles represent mRNAs.

According to the cumulative somatic mutation count of each sample, there were 103 samples classified in the GU group with the top 25% mutation frequencies and 109 samples in the GS group with the bottom 25% mutation frequencies. A total of 155 lncRNAs with differential expression between the GU group and the GS group were recognized as genome instability-associated lncRNAs, among which 90 lncRNAs were upregulated and 65 lncRNAs were downregulated in the GU group (fold change greater than 2 or less than 0.5, FDR-adjusted *P* value < 0.05, Wilcoxon test, Supplementary Table 1). The top 20 lncRNAs with the most upregulation and downregulation were selected to draw a heat map (Figure 2A). Based on the expression levels of 155 genome instability-related lncRNAs, all patients were arranged into two clusters, the GS-like cluster and the GU-like cluster (Figure 2B). The somatic mutation frequency was significantly higher in the GU-like cluster than in the GS-like cluster (*P* value < 0.001, Mann-Whitney *U* test, Figure 2C).

### Co-expression Network Construction and Functional Enrichment Analysis of Genome Instability-Related Genes

**FIGURE 3:**
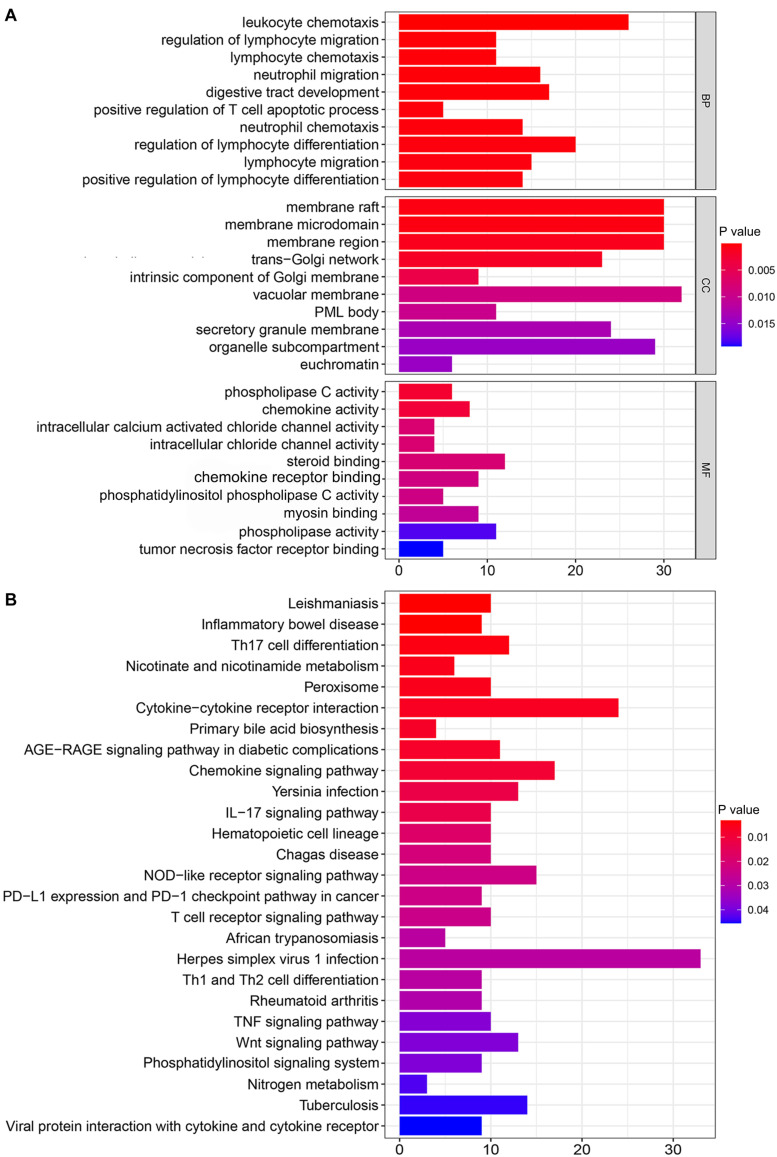
Functional enrichment analysis of mRNAs co-expressed with genome instability-associated lncRNAs. **(A)** Gene Ontology (GO) analysis of biological processes (BPs), cellular components (CCs), and molecular functions (MFs). **(B)** Kyoto Encyclopedia of Genes and Genomes (KEGG) pathway analysis.

The top 10 mRNAs most correlated with each lncRNA were selected as target genes. After linking the correlated mRNAs and lncRNAs together, a lncRNA-mRNA co-expression network was constructed for visualization (Figure 2D). GO analysis revealed that the top 10 enrichment terms of the identified mRNAs were mainly involved in the chemotaxis and migration of leukocytes, such as lymphocytes and neutrophils for biological processes (BPs) (*P* value < 0.001, Figure 3A); organelle subcompartment, pro-myelocytic leukemia protein (PML) body and euchromatin for cellular components (CCs) (*P* value < 0.015, Figure 3A); and phospholipase C activity and tumor necrosis factor (TNF) receptor binding for molecular functions (MFs) (*P* value < 0.020, Figure 3A). The results of KEGG pathway analysis indicated that the target genes were mainly enriched in T cell differentiation, TNF signaling pathway, Wnt signaling pathway, phosphatidylinositol signaling system, nicotinate and nicotinamide metabolism, peroxisome, primary bile acid biosynthesis, and nitrogen metabolism (*P* value < 0.05, Figure 3B). GO and KEGG analyses showed that these mRNAs were associated with immunity, inflammation, bile acid biosynthesis, carcinogenesis and genome instability. Therefore, the 155 differentially expressed lncRNAs could act as candidate genome instability-related lncRNAs in colon cancer. Immune system might involve in the genome instability and carcinogenesis of colon cancer.

### Development of a Genome Instability-Associated LncRNA Signature as Prognostic Models

**FIGURE 4:**
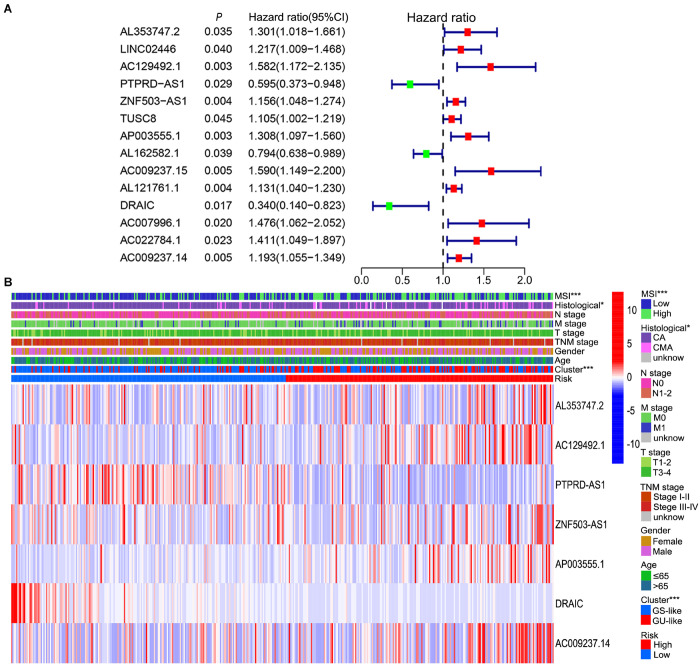
Prognosis-related lncRNAs and correlation of prognostic risk score with clinicopathological features. **(A)** Forest plot of 14 prognosis-related lncRNAs after univariate Cox regression analysis in training set. The red points represent risk lncRNAs with a mean hazard ratio larger than 1, while the green points represent protective lncRNAs with a mean hazard ratio less than 1. The blue bar indicates the 95% CI of the hazard ratio. **(B)** Relationship among prognostic risk, MSI, genome instability cluster, clinicopathological characteristics and expression level of seven lncRNAs in the signature in TCGA set. CI, confidence interval; MSI, microsatellite instability; CA, colon adenocarcinoma; CMA, colon mucinous adenocarcinoma; GS-like, genome stable-like; GU-like, genome unstable-like; *P value < 0.05; ***P value < 0.001.

To further explore the prognostic role of these candidate genome instability-related lncRNAs, all samples from the TCGA database were randomly divided into a training set (*n* = 210) and a testing set (*n* = 208). The baseline clinical information, including age, sex and TNM stage, of the patients in the training set and testing set were comparable (*P* value > 0.05, chi-square test, Supplementary Table 2). In the training set, 14 prognosis-related lncRNAs were identified from the 155 genome instability-associated lncRNAs using univariate Cox analysis, and the forest plot of these lncRNAs is shown in Figure 4A. Given that 7 of 14 candidate lncRNAs still retained prognostic value in multivariate Cox analysis, they were selected as independent prognostic lncRNAs and included in the lncSig finally (Table 1). The risk score calculation formula for the OS of each patient was the cumulative sum of the coefficient multiplied by the expression level of each lncRNA in the genome instability-associated signature as follows: (0.40 × expression level of AL353747.2) + (0.42 × expression level of AC129492.1) + (0.18 × expression level of ZNF503-AS1) + (0.28 × expression level of AP003555.1) + (0.16 × expression level of AC009237.14) + (−1.00 × expression level of DRAIC) + (−0.52 × expression level of PTPRD-AS1). LncRNAs with HR > 1 (positive coefficient) represented a high risk for OS, and lncRNAs with HR < 1 (negative coefficient) indicated a low survival risk, suggesting that AL353747.2, AC129492.1, ZNF503-AS1, AP003555.1 and AC009237.1 might function as risk factors for clinical outcome, while DRAIC and PTPRD-AS1 could be protective factors, with high expression associated with better prognosis. Furthermore, the expression levels of these lncRNAs were compared between tumor and normal tissue, indicating AL353747.2, AC129492.1, AP003555.1 up-regulated, while DRAIC down-regulated in colon cancer than normal tissue (Supplementary Figure 1).

**TABLE 1:** Multivariate Cox regression analysis of the seven lncRNAs in lncRNA signature.

ID	Coefficient	HR	HR.95%Low	HR.95%High	*P* value
AL353747.2	0.399905	1.491682	1.066592	2.086193	0.019457
AC129492.1	0.418519	1.51971	1.09246	2.114052	0.012953
PTPRD-AS1	−0.52315	0.592651	0.35915	0.977962	0.040642
ZNF503-AS1	0.182342	1.200025	1.095536	1.314479	8.74E-05
AP003555.1	0.276715	1.318791	1.073708	1.619816	0.008341
DRAIC	−0.99856	0.36841	0.143494	0.945864	0.037926
AC009237.14	0.164247	1.178505	1.018323	1.363884	0.027555

*HR, hazard ratio.*

### Relationship of Genome Instability With Prognosis and Immune Infiltration Based on LncSig

**FIGURE 5:**
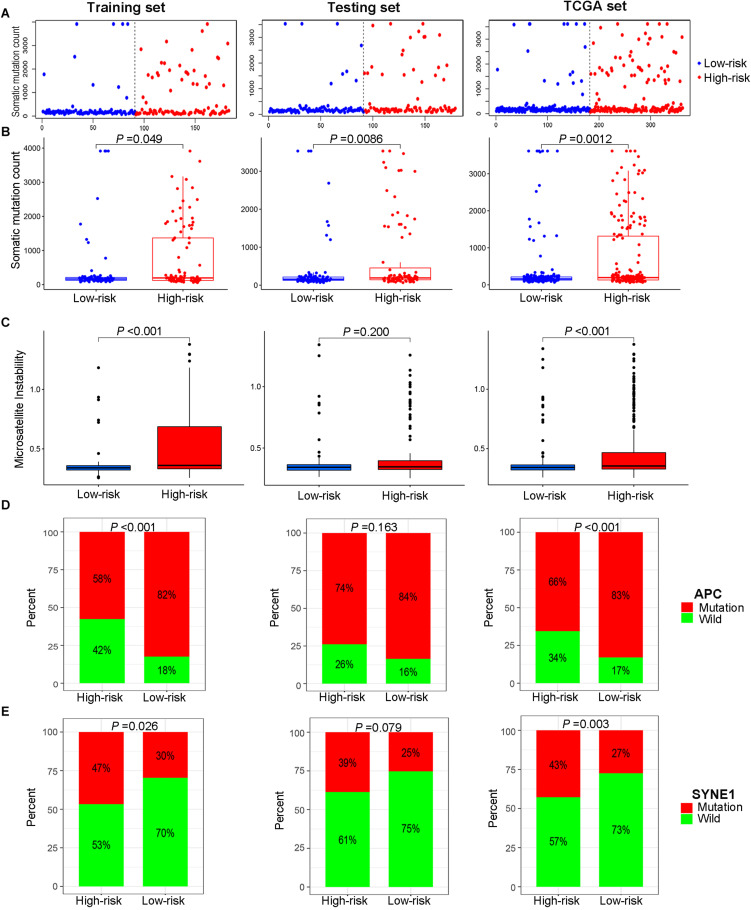
Relationship between genome instability and the risk score calculated by the lncRNA signature. **(A)** Distribution of somatic mutation count of patients sorted by increasing risk score. **(B)** Cumulative somatic mutation number of patients in the high-risk group and low-risk group. **(C)** Microsatellite instability score of patients in high- and low-risk groups. **(D)** Proportion of APC mutations in the high- and low-risk groups. **(E)** Proportion of SYNE1 mutations in the high- and low-risk groups. All illustrations are shown in the sequence of the training set, testing set and TCGA set.

**FIGURE 6:**
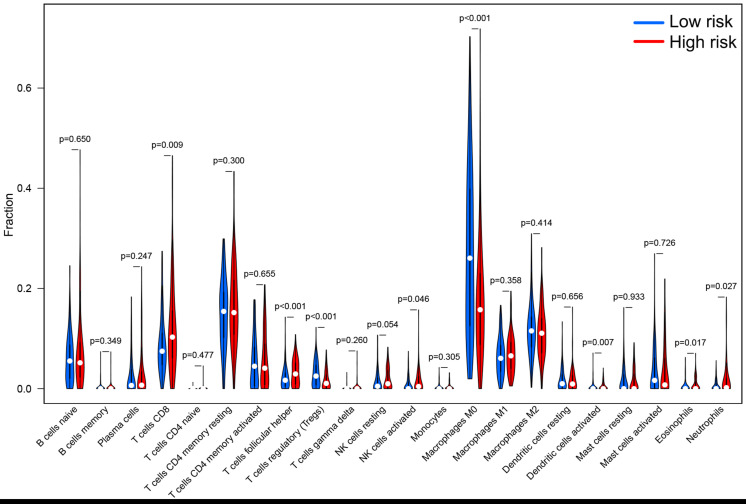
The differential infiltrating levels of 22 immune cell types in high-risk group and low-risk group. Patients with high prognostic risk showed larger infiltration of T cells CD_8_^+^, T cells follicular helper, NK cells activated, dendritic cells activated, eosinophils and neutrophils, while low risk score correlated with more infiltration of T cells regulatory (Treg) and macrophages M0. *P* value < 0.05 was regarded as a statistical criterion.

According to the median of the risk score in the training set as the cutoff point, the patients in the training set were evenly divided into a high-risk group (*n* = 105) and a low-risk group (*n* = 105). As for the testing set, 208 patients were assigned to the high-risk group (*n* = 101) and the low-risk group (*n* = 107). In summary, a total of 418 patients were classified into the high-risk group (*n* = 206) and the low-risk group (*n* = 212). Patients with high MSI, GU-like cluster and colon mucinous adenocarcinoma tended to have high prognostic risk (*P* value < 0.05, chi-square test, Figure 4B). Scatter plots of somatic mutation count for samples sorted by increasing order of risk score were illustrated (Figure 5A). The somatic mutation count was verified to be higher in the high-risk group than in the low-risk group among the training, testing and TCGA sets (*P* value < 0.05, Mann-Whitney *U* test, Figure 5B). MSI in high-risk group was higher than low-risk group in training set and entire TCGA set (*P* value < 0.05, Mann-Whitney *U* test, Figure 5C). The top 6 genes with the highest mutation frequencies among all patients were APC, TTN, TP53, KRAS, MUC16, and SYNE1. Further analyses for the correlation of the mutation status of a single gene with the prognostic risk were performed, and the proportion of APC mutations was larger in low-risk samples (*P* value < 0.001, chi-square test), while the proportion of SYNE1 mutations was larger in high-risk samples (*P* value < 0.05, chi-square test) in the training set and TCGA set (Figures 5D,E). All these results indicated the patients in high-risk group characterized by genome instability status. As for the fraction of 22 infiltrated immune cell types, low risk score correlated with more infiltration of T cells regulatory (Treg) and macrophages M0, while high-risk group showed larger infiltration of T cells CD_8_^+^, T cells follicular helper, NK cells activated, dendritic cells activated, eosinophils and neutrophils (*P* value < 0.05, Mann-Whitney *U* test, Figure 6). In a word, alternation of immune infiltration, as one of the characteristics of tumor, might correlate with genome instability in colon cancer.

### Validation of Genome Instability-Associated LncSig as Prognostic Model

**FIGURE 7:**
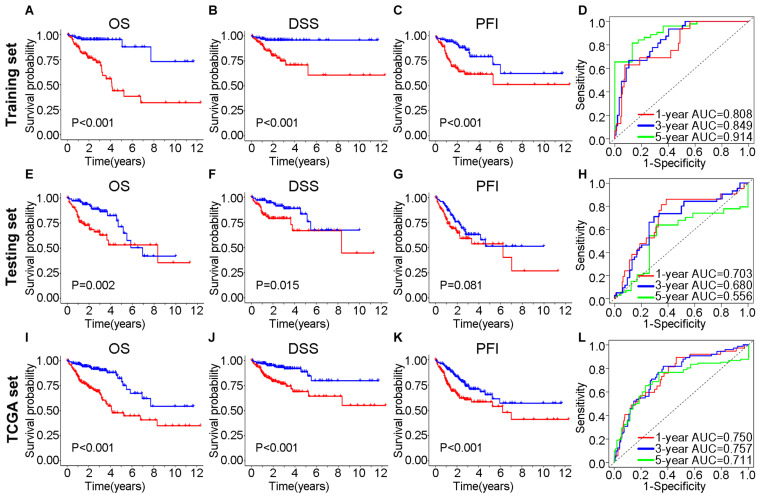
Efficiency validation of the genome instability-associated lncRNA signature for prognostic prediction. In training set, Kaplan–Meier curves for OS **(A)**, DSS **(B)** and PFI **(C)** of patients and the receiver operating characteristic (ROC) curves **(D)**. In testing set, impact of prognostic risk on OS **(E)**, DSS **(F)** and PFI **(G)** of patients and the ROC curves **(H)**. In TCGA set, impact of prognostic risk on OS **(I)**, DSS **(J),** and PFI **(K)** of patients and the ROC curves **(L)**. OS, overall survival; DSS, disease-specific survival; PFI, progression-free interval; AUC, area under the curve.

**FIGURE 8:**
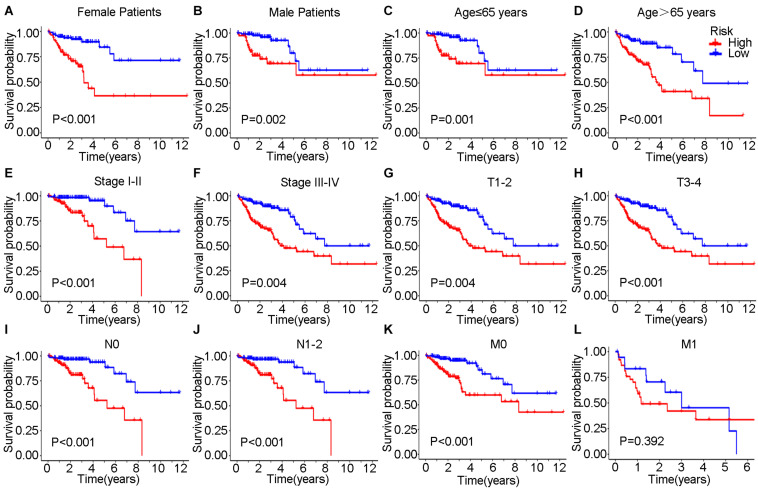
Kaplan–Meier curves were performed for patients stratified by clinicopathological features in the TCGA set. Impact of prognostic risk on overall survival for female **(A)** and male patients **(B)**; for patients younger than 65 years old **(C)** and older than 65 years old **(D)**; for patients in stage I-II **(E)** and stage III-IV **(F)**; for patients in stage T1-2 **(G)** and stage T3-4 **(H)**; for patients in stage N0 **(I)** and stage N1-2 **(J)**; and for patients in stage M0 **(K)** and stage M1 **(L)**.

In the training set, Kaplan–Meier curves showed that the OS, DSS, and PFI in the high-risk group were much poorer than low-risk group (*P* value < 0.001, log-rank test, Figures 7A–C). The ROC curves yielded 1-year, 3-year and 5-year AUCs of 0.808, 0.849, and 0.914 for lncSig in the training set, respectively (Figure 7D). As for testing set, patients in the high-risk group still had a shorter OS (*P* value = 0.002) and DSS (*P* value = 0.015) than those in the low-risk group (Figures 7E–G). The 1-year, 3-year and 5-year AUCs of lncSig were 0.703, 0.680, and 0.556 in the testing set, respectively (Figure 7H). The survival analysis results in the TCGA set were consistent with those in the training set (*P* value < 0.001, log-rank test, Figures 7I–K). The 1-year, 3-year, and 5-year AUCs of lncSig were 0.750, 0.757, and 0.711 in the TCGA set, respectively (Figure 7L). In summary, lncSig could effectively evaluate the prognosis of colon cancer. To further explore the predictive effect of lncSig for patients with different clinical factors in the TCGA set, stratification analyses according to sex, age and stage were performed with Kaplan–Meier curves. As a result, except for stage M1 patients, the OS of the high-risk group was worse than that of the low-risk group in all the other subgroups, including males and females, age ≤ 65 years and age > 65 years, stage I-II and stage III-IV, stage T1-T2, and stage T3-T4, stage N0 and stage N1-N2 and stage M0 (*P* value < 0.05), suggesting that lncSig could be applied to all male and female, young and elderly, early and advanced colon cancer patients as a prognostic indictor (Figure 8). Next, univariate and multivariate Cox regression analyses were conducted to clarify whether risk score of lncSig was independent among common clinicopathological variables in TCGA set. In univariate Cox regression analysis, age (*P* value = 0.048), T stage (*P* value < 0.001), M stage (*P* value < 0.001), N stage (*P* value < 0.001) and risk score (*P* value < 0.001) were correlated with the OS. After enrolling these factors in multivariate Cox analysis, T stage (*P* value = 0.004), M stage (*P* value = 0.001), N stage (*P* value = 0.004) and risk score (*P* value < 0.001) were still associated with OS independently (Table 2). In a word, risk score calculated by genome instability-associated LncSig could act as an independent prognostic factor for colon cancer patients in TCGA cohort.

**TABLE 2:** Cox regression analyses of variables with overall survival in TCGA set.

Variables		Univariate analysis			Multivariate analysis		
		HR	95% CI	*P*	HR	95%CI	*P*
Age	>65/≤65	1.023	1.000–1.046	0.048	1.022	1.000–1.045	0.053
Gender	Female/Male	1.046	0.630–1.736	0.861			
T stage	T3-4/T1-2	3.711	2.220–6.201	<0.001	2.406	1.325–4.370	0.004
M stage	M1/M0	6.125	3.635–10.322	<0.001	2.810	1.518–5.200	0.001
N stage	N1-2/N0	2.339	1.734–3.155	<0.001	1.678	1.176–2.394	0.004
Risk Score	High/Low	1.155	1.095–1.218	<0.001	1.128	1.064–1.196	<0.001

*HR, hazard ratio; CI, confidence interval.*

### Independent Validation and Comparison of LncSig With Other LncRNA Signatures

**FIGURE 9:**
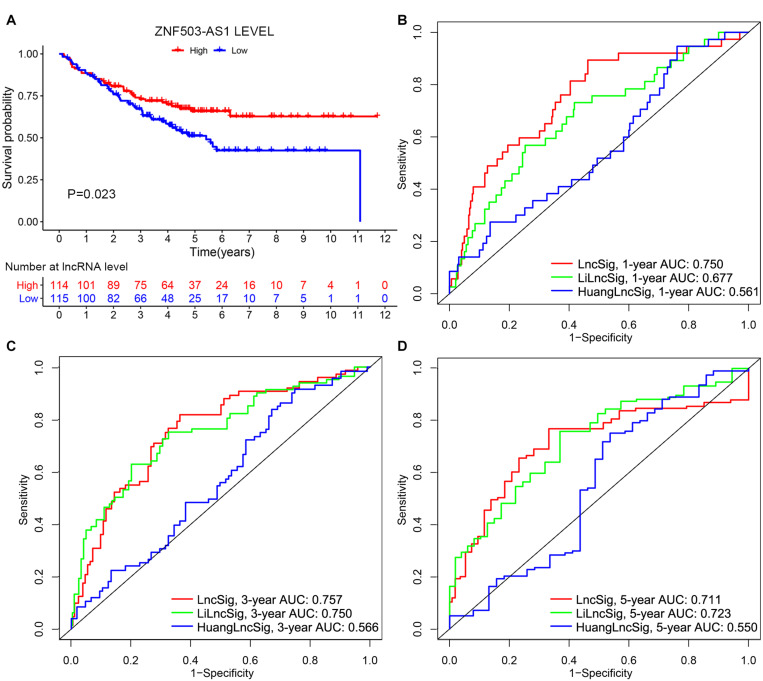
Performance evaluation of lncRNA signature. **(A)** Independent validation of the lncRNA in lncRNA signature in the GSE17538 dataset. Kaplan–Meier curves illustrated that patients with low expression levels of ZNF503-AS1 had worse overall survival than those with high expression levels (*P* value = 0.023). 1-year **(B)**, 3-year **(C),** and 5-year **(D)** receiver operating characteristic (ROC) analyses for our lncRNA signature (lncSig), Li lncRNA signature (LiLncSig) and Huang lncRNA signature (HuangLncSig). AUC, area under the curve.

To further examine the prognostic value of lncSig, the lncRNA ZNF503-AS1, as the only lncRNA from lncSig that could be found in GSE17538, was utilized to predict the clinical outcome of patients with colon cancer. Patients with low expression levels of ZNF503-AS1 exhibited poorer OS than those with high levels, indicating that ZNF503-AS1 might play a protective role in the prognosis of colon cancer (*P* value = 0.023, Figure 9A). Several researchers have recently published lncRNA signatures for prognosis prediction in colon cancer. Li proposed a lncRNA signature, including AC027307.2, AC074117.1, AC103702.2, CYTOR, LINC02381, MIR200CHG, and SNHG16 (Li Z. et al., 2020). Huang developed a predictive signature including XXbac-B476C20.9, PP7080, CDKN2B-AS1, LINC00092, CA3-AS1, HAND2-AS1, CTD-2269F5.1, and LINC01082 (Huang et al., 2019). The risk scores calculated according to the expression levels of these lncRNAs in the two models above were applied to TCGA cohort. According to ROC curve analyses, and the 1-year AUCs of HuangLncSig, LiLncSig and our lncSig were 0.561, 0.677, and 0.750, the 3-year AUCs were 0.566, 0.750, and 0.757, and the 5-year AUCs were 0.550, 0.723, and 0.711, respectively (Figures 9B–D), indicating that our lncSig tended to have better performance for 1-year and 3-year OS prediction than these two previously published lncRNA signatures.

## Discussion

Accumulating evidence has revealed that genome instability is one of the ubiquitous hallmarks of most cancers involved in tumorigenesis and progression (Bartkova et al., 2005; Negrini et al., 2010). Genomic instability arises from many different pathways, including telomere damage, epigenetic modifications, and DNA damage, ranging from simple gene mutations to abnormalities of chromosomes (Ferguson et al., 2015). The genome sequencing of multiple kinds of cancers has revealed repertoire somatic mutational signatures for the underlying biological processes in cancer development (The ICGC/TCGA Pan-Cancer Analysis of Whole Genomes Consortium, 2020; Alexandrov et al., 2020). Recently, mutation burden has been discovered to substantially increase in colorectal cancer compared with normal cells (Lee-Six et al., 2019), and to be significantly associated with survival outcomes and the objective response to immune therapy (Domingo et al., 2018; Schrock et al., 2019; Wang et al., 2020). Similarly, MSI, an attractive target for immune checkpoint inhibitors, could correlate with survival outcomes (Hause et al., 2016). Therefore, the degree of genomic instability could have valuable applicability for diagnostic, therapeutic and prognostic prediction. Compared with other mutation calling methods, MuTect is particularly useful for studying genome sequencing data with a low mutated allelic fraction because of its higher sensitivity and similar specificity (Cibulskis et al., 2013). However, it is still a challenge to quantify the somatic mutation burden with portable methods in clinical practice.

In recent studies, lncRNAs have become a growing focus for cancer genomics studies with crucial regulatory functions in multiple biological processes in cancers, indicating that lncRNAs could exhibit potential as biomarker candidates for carcinogenesis and prognosis (Gupta et al., 2010; Prensner et al., 2011; Bhan et al., 2017; Peng et al., 2017). Two lncRNAs, MIR4458HG, and LINC01235, showed significant prognostic value for stomach adenocarcinoma (Zhang X. et al., 2020). Gao Y. et al. (2019) characterized some lncRNAs with expression levels affected by somatic mutations across 17 cancer types and validated them as survival indicators. Recently, several lncRNAs, such as NORAD (Munschauer et al., 2018) and GUARDIN (Hu et al., 2018), have been recognized as essential for genomic stability. However, the identification of genome instability-related lncRNAs and their prognostic value is still largely unexplored. Therefore, we obtained 155 lncRNAs with different expression levels in the 25% highest and 25% lowest mutation frequency samples, and these lncRNAs were characterized as genome instability-associated lncRNAs. The results of functional enrichment analyses of the mRNAs co-expressed with the 155 lncRNAs indicated those lncRNA might play important roles in the tumorigenesis, genome instability and immune system of colon cancer, which were consistent with other studies. For instance, neutrophils from patients with cancer exhibited not only more spontaneous migration but also distinguishable metabolic patterns relative to control neutrophils (Patel et al., 2018). Tumor-associated neutrophils recruited macrophages and T-regulatory lymphocytes to promote progression and therapeutic resistance in hepatocellular carcinoma (Zhou et al., 2016) and might induce genome instability to impede resolution of intestinal inflammation and wound healing (Butin-Israeli et al., 2019). The PML body was reported to act as a tumor suppressor by maintaining genome stability (Nagai et al., 2011). As critical steps in cancer-related biological processes, phospholipase C genes (Waugh, 2016), the TNF signaling pathway (Heijink et al., 2019), the Wnt signaling pathway (Augustin et al., 2017), phosphatidylinositol signaling systems such as the PI3K-AKT pathway (Hu et al., 2020), and peroxisome proliferator-activated receptors (Li et al., 2019) are also involved in genome stability. Bile acids are metabolized by intestinal bacteria and associated with gastrointestinal tumorigenesis in colorectal cancer and hepatocellular carcinoma (Jia et al., 2018), but the correlation between bile acids and genome stability still needs further exploration. In summary, the enrichment of co-expressed genes was associated with carcinogenesis, genome stability and immune, and these lncRNAs could be used to evaluate genome instability in colon cancer.

It was validated that the seven lncRNAs in lncSig were not only indicators of genome instability but also predictors of the clinical outcomes of cancer patients in this study. The significant differences in OS, DSS, and PFI exhibited between the high-risk and low-risk groups suggested that lncSig has prognostic value for all TCGA patients as well as patients in subgroups with different clinical statuses except M1 stage. We highlighted that lncSig acted as an independent prognostic indicator for all patients in TCGA cohort and had a good performance of OS prediction, which was confirmed by Cox regression analysis and ROC curves. These results elucidate that the genome instability-associated lncSig might have prognostic significance for colon cancer. The biological functions of 3 lncRNAs in our lncSig have been studied recently. ZNF503-AS1 was identified to promote retinal pigment epithelium differentiation (Chen et al., 2017) and act as a tumor suppressor in bladder cancer (He et al., 2020). In this study, ZNF503-AS1 tended to be a risk factor with a positive coefficient in lncSig, while the OS of patients with low ZNF503-AS1 expression was worse than those patients with high ZNF503-AS1 expression in the GSE17538. Considering that the role of this lncRNA has not been investigated in colon cancer before this study, further experiments are essential to validate the effect of ZNF503-AS1 on colon cancer prognosis. DRAIC could inhibit the progression of prostate cancer (Saha et al., 2020) and gastric cancer (Zhang Z. et al., 2020), facilitate the proliferation and migration of nasopharyngeal carcinoma (Liao et al., 2019) and regulate autophagic flux (Tiessen et al., 2019). PTPRD-AS1 had potential as a prognostic predictor and was included in an immunotherapeutic response lncRNA signature for bladder cancer (Gao X. et al., 2019; Wu et al., 2020). However, none of the 7 lncRNAs have been found to be related to genome stability before, and our research was the first study to reveal the potential of these 7 lncRNAs as genome instability and prognostic indicators for colon cancer, which deserve further investigation in the future.

In this study, APC, TTN, TP53, KRAS, MUC16, and SYNE1 were discovered as top 6 most mutated genes in colon cancer. APC, associated with the dysregulation of the Wnt signaling pathway, was recognized as the early driver gene mutated in both colon adenomas and carcinomas (Wolff et al., 2018). Schell et al. (2016) revealed that APC played a central role in predicting the OS of colorectal cancer. The prognosis of patients without APC mutations was worse than that of patients with single APC mutations (Schell et al., 2016), which is consistent with our finding that the proportion of wild-type APC was larger in high-risk samples in the training set and TCGA set. We also demonstrated that the proportion of SYNE1 mutations was larger in patients with high prognostic risk in the training set and TCGA set. A similar result was found that the presence of concurrent mutations of SYNE1 and TTN not only led to worse clinical outcomes but was also related to the response to drug treatment in colorectal cancer (Zhou et al., 2020). Through analysis of the percentage of prognosis-related gene mutations, lncSig could be recognized as a promising predictor of clinical outcomes in colon cancer.

Some researchers have found that lncRNAs could serve as important regulators of immune response and potential biomarkers for cancer patients (Peng et al., 2019; Tamang et al., 2019; Zhao et al., 2019). Based on the enrichment analysis in this study, some immune signaling pathway might associate with genome instability. Correspondingly, our results showed that the two risk groups expressed differential genome stability status and infiltrated immune cell subtypes. Immunotherapy is reported effective for metastatic DNA mismatch repair-deficient colorectal cancer with high MSI that demonstrate immune infiltration (Overman et al., 2017; Lee et al., 2020). Genome instability and tumor mutation might cause abundant neoantigens, then numerous T cell would recognize the neoantigens and contribute to large immune cell infiltration. Above all, genome instability correlated with immune infiltration and prognosis of colon cancer patients. Distinguishing the genome instability and immune infiltration of colon cancer might benefit patients from anti-tumor immunotherapy.

Although we provided a new understanding in correlating genome instability, immune infiltration and clinical outcomes through lncRNAs, there are still some limitations in our study. Firstly, given that many microarrays were not designed for lncRNAs, we could hardly find suitable cohorts which include lncRNA expression and clinical survival data at the same time to validate our prognostic signature. Large-scale datasets and clinical samples should be used to validate the robustness of the seven-lncRNA signature and ZNF503-AS1 as prognostic indicators. Moreover, the biological functions of the genome instability-associated lncRNAs should be explored by further *in vitro* and *in vivo* studies to explain the mechanisms involved in genome stability regulation and the carcinogenesis of colon cancer in the future. Finally, whether these 7 lncRNAs combined with other clinical factors could exhibit better capability for prognostic prediction remains an interesting issue and requires further validation.

In summary, we identified genome instability-associated lncRNAs and performed functional enrichment analysis, which may assist in understanding the crucial role of lncRNAs in genome stability and immune cell infiltration in colon cancer. A genome instability-related lncRNA signature was proposed for clinical outcome prediction, and its efficiency was verified successfully. Along with comprehensive experimental studies for further validation later, the lncRNA signature may provide new opportunities for improving clinical outcome prediction and guiding personalized treatment as a genome instability and prognosis biomarker.

## Data Availability Statement

The publicly available datasets are analyzed in this study. This data can be found here: The Cancer Genome Atlas (TCGA) database (https://portal.gdc.cancer.gov/) and Gene Expression Omnibus (GEO) database (https://www.ncbi.nlm.nih.gov/geo/).

## Ethics Statement

The studies involving human participants were reviewed and approved by the Clinical Research Ethics Committee of China-Japan Friendship Hospital. Written informed consent for participation was not required for this study in accordance with the national legislation and the institutional requirements.

## Author Contributions

TY conceived and designed the study, performed formal analysis, prepared the original draft, and reviewed this manuscript. DZ participated in data analysis and manuscript revision critically. SY designed and supervised the study, revised the manuscript, and obtained the funding. All authors read and approved the final manuscript.

## Conflict of Interest

The authors declare that the research was conducted in the absence of any commercial or financial relationships that could be construed as a potential conflict of interest.

## Footnotes

1 http://portal.gdc.cancer.gov

2 https://www.ncbi.nlm.nih.gov/geo/

3 https://www.gencodegenes.org
