# Supplementary material for: Identification of a Genome Instability-Associated LncRNA Signature for Prognosis Prediction in Colon Cancer
Source: Front Genet. 2021 Jun 7;12:679150. doi: 10.3389/fgene.2021.679150 (PMC8215581; doi:10.3389/fgene.2021.679150)
Supplement: Supplementary file 1 [file Image_1.pdf]

Supplementary Material

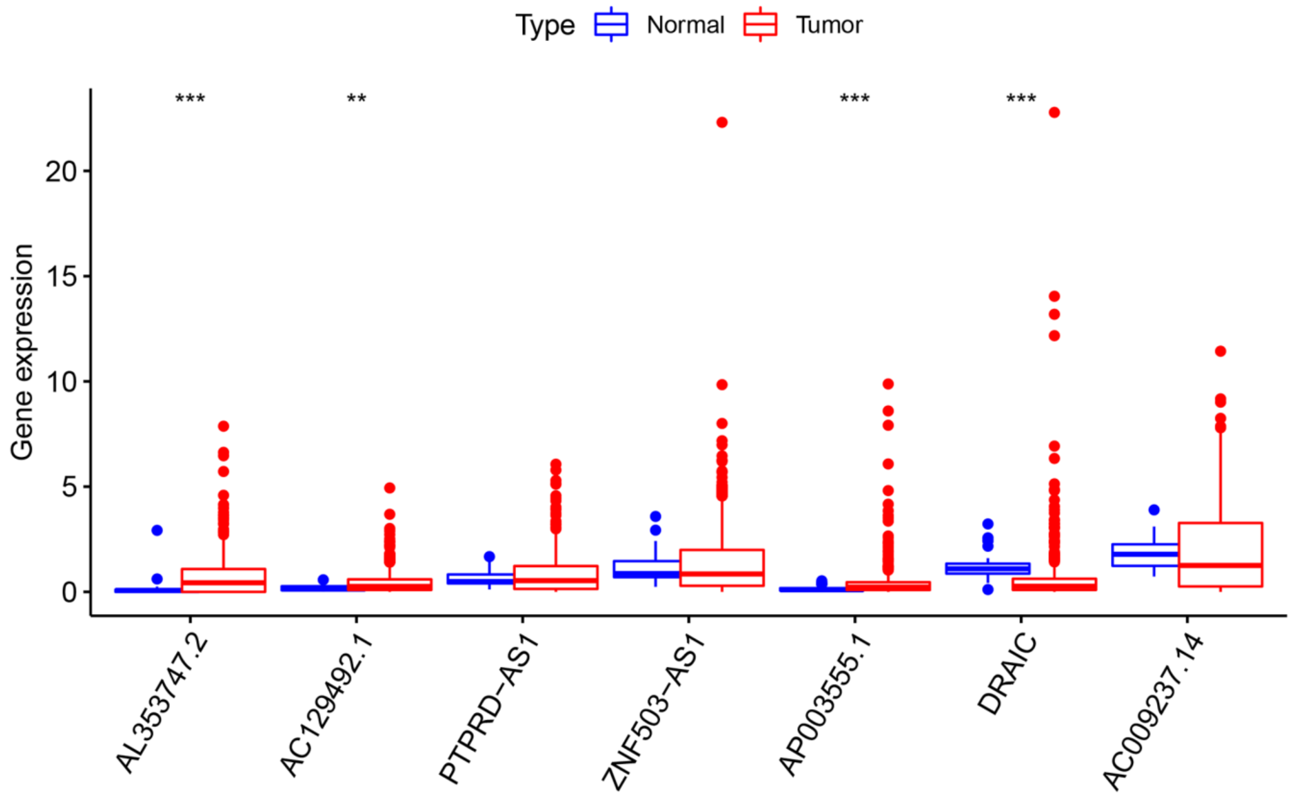

FIGURE S1 | Expression level of lncRNAs in signature in tumor and normal tissue. **, *P* value <0.01; ***, *P* value <0.001.
