# Supplementary material for: Identification of a Genome Instability-Associated LncRNA Signature for Prognosis Prediction in Colon Cancer
Source: Front Genet. 2021 Jun 7;12:679150. doi: 10.3389/fgene.2021.679150 (PMC8215581; doi:10.3389/fgene.2021.679150)
Supplement: Supplementary file 2 [file Table_1.DOCX]

Supplementary Material

**TABLE S1 |** 155 lncRNAs related to genome instabiltiy of colon cancer

| **LncRNA** | **GU group** | **GS group** | **logFC** | ***P* value** | **Adjusted *P* value** |
| --- | --- | --- | --- | --- | --- |
| AC108865.1 | 7.797117 | 0.5146629 | -3.92124 | 5.69E-08 | 7.68E-07 |
| AC108865.2 | 3.0555485 | 0.2898963 | -3.39782 | 4.77E-06 | 3.36E-05 |
| AC026336.3 | 1.3759916 | 0.2087364 | -2.72072 | 1.33E-11 | 5.38E-10 |
| AC135388.1 | 0.7193871 | 0.1175793 | -2.61313 | 8.37E-15 | 1.12E-12 |
| AC078993.1 | 4.7282977 | 0.7863573 | -2.58806 | 3.53E-11 | 1.16E-09 |
| AC010280.1 | 1.1260654 | 0.1980654 | -2.50724 | 2.41E-15 | 3.82E-13 |
| AC136475.9 | 0.683536 | 0.13801 | -2.30824 | 2.51E-06 | 1.92E-05 |
| AC114296.1 | 0.8662932 | 0.1871049 | -2.21101 | 2.35E-13 | 1.70E-11 |
| LINC01811 | 1.0583565 | 0.2549984 | -2.05327 | 2.74E-11 | 9.35E-10 |
| AC110772.2 | 1.0326937 | 0.2488552 | -2.05303 | 0.000144 | 0.000647 |
| LINC00654 | 1.6673342 | 0.4391156 | -1.92487 | 4.13E-13 | 2.77E-11 |
| LINC02563 | 1.2174576 | 0.3252451 | -1.90427 | 6.77E-11 | 1.96E-09 |
| BOK-AS1 | 2.403557 | 0.662026 | -1.86021 | 0.00349 | 0.009392 |
| AC093585.1 | 0.7326316 | 0.2043131 | -1.84231 | 1.36E-11 | 5.38E-10 |
| AP003774.2 | 17.028648 | 4.7905333 | -1.82971 | 1.37E-16 | 4.78E-14 |
| AP005271.1 | 0.9300564 | 0.2785075 | -1.7396 | 1.21E-09 | 2.56E-08 |
| TUSC8 | 3.948341 | 1.2192121 | -1.6953 | 3.78E-15 | 5.49E-13 |
| LINC00525 | 0.8123438 | 0.2516718 | -1.69055 | 1.60E-14 | 1.85E-12 |
| PTPRD-AS1 | 1.0767826 | 0.335852 | -1.68083 | 2.00E-14 | 2.18E-12 |
| AC116345.1 | 1.0083619 | 0.3171944 | -1.66857 | 1.29E-05 | 7.97E-05 |
| AC053503.3 | 2.3189023 | 0.7332213 | -1.66112 | 0.016829 | 0.034921 |
| AC108134.3 | 2.7904113 | 0.9083341 | -1.61918 | 3.08E-10 | 7.34E-09 |
| AL117382.2 | 12.852154 | 4.2354386 | -1.60143 | 9.15E-17 | 4.55E-14 |
| AC017074.1 | 3.230957 | 1.112035 | -1.53876 | 6.36E-19 | 1.11E-15 |
| LINC01819 | 5.4028356 | 1.8642188 | -1.53515 | 0.00036 | 0.00136 |
| AP004608.1 | 1.4728118 | 0.5090804 | -1.53261 | 1.74E-13 | 1.37E-11 |
| AL121832.1 | 1.0211825 | 0.3599423 | -1.5044 | 5.11E-09 | 9.68E-08 |
| CHN2-AS1 | 0.832345 | 0.2991146 | -1.47648 | 8.59E-09 | 1.51E-07 |
| LINC02418 | 5.222422 | 1.893768 | -1.46346 | 2.51E-11 | 8.74E-10 |
| AC055717.2 | 1.8694859 | 0.6817827 | -1.45526 | 1.32E-11 | 5.38E-10 |
| AL121895.2 | 0.865571 | 0.3172987 | -1.44781 | 4.20E-11 | 1.33E-09 |
| AC123023.1 | 2.1961678 | 0.8267252 | -1.40951 | 5.12E-13 | 3.24E-11 |
| LINC02441 | 3.5718358 | 1.3512968 | -1.40232 | 6.13E-13 | 3.56E-11 |
| SATB2-AS1 | 3.4088746 | 1.2917788 | -1.39994 | 3.27E-13 | 2.28E-11 |
| OSER1-DT | 5.4274584 | 2.1265817 | -1.35174 | 3.57E-18 | 3.11E-15 |
| SMIM2-AS1 | 1.8177023 | 0.7128688 | -1.35041 | 1.24E-09 | 2.60E-08 |
| LHFPL3-AS2 | 3.0873327 | 1.221049 | -1.33824 | 0.000197 | 0.000834 |
| AL121829.2 | 0.7265887 | 0.2945562 | -1.3026 | 7.07E-07 | 6.38E-06 |
| HNF4A-AS1 | 1.368755 | 0.5566478 | -1.29803 | 0.000746 | 0.002583 |
| AC124067.4 | 11.535329 | 4.7020389 | -1.2947 | 3.59E-16 | 8.56E-14 |
| ZNF503-AS1 | 1.7368915 | 0.7088117 | -1.29303 | 2.23E-07 | 2.41E-06 |
| CFTR-AS1 | 1.0226956 | 0.417928 | -1.29105 | 4.34E-06 | 3.09E-05 |
| AC109446.3 | 2.1264478 | 0.8752511 | -1.28068 | 1.06E-10 | 2.94E-09 |
| AL162582.1 | 1.8458558 | 0.7630591 | -1.27442 | 1.01E-05 | 6.50E-05 |
| LINC02487 | 1.310179 | 0.5428033 | -1.27126 | 1.05E-12 | 5.91E-11 |
| AC009812.1 | 1.4362727 | 0.5959737 | -1.26901 | 1.52E-13 | 1.26E-11 |
| AC254629.1 | 4.7909103 | 2.0051339 | -1.2566 | 5.74E-13 | 3.45E-11 |
| AC090579.1 | 0.8197711 | 0.3447308 | -1.24975 | 1.74E-11 | 6.60E-10 |
| AL133370.1 | 9.0970769 | 3.8370393 | -1.24541 | 2.77E-05 | 0.000156 |
| DPP10-AS1 | 1.1190447 | 0.4737126 | -1.24018 | 0.00135 | 0.004235 |
| AC009237.14 | 2.4562294 | 1.0409043 | -1.23861 | 2.21E-11 | 8.03E-10 |
| DIO3OS | 3.1628975 | 1.3455241 | -1.23308 | 1.29E-11 | 5.38E-10 |
| AC105219.1 | 1.0894379 | 0.46559 | -1.22645 | 0.000505 | 0.001837 |
| AL590483.1 | 0.6922017 | 0.3002973 | -1.2048 | 2.37E-09 | 4.80E-08 |
| AC064807.2 | 0.8177847 | 0.355602 | -1.20146 | 4.38E-07 | 4.24E-06 |
| AC106876.1 | 8.1681396 | 3.6276021 | -1.17099 | 3.93E-16 | 8.56E-14 |
| AC090709.1 | 0.5811113 | 0.2584241 | -1.16907 | 0.00036 | 0.00136 |
| AL022313.2 | 1.0829569 | 0.481974 | -1.16795 | 1.08E-06 | 9.29E-06 |
| AL365226.1 | 39.781928 | 17.969828 | -1.14654 | 6.80E-05 | 0.000335 |
| LINC01006 | 3.6128109 | 1.6446253 | -1.13536 | 1.04E-16 | 4.55E-14 |
| LINC02747 | 6.8055536 | 3.1172646 | -1.12643 | 1.17E-10 | 3.15E-09 |
| AL353747.2 | 0.7569319 | 0.3475327 | -1.12301 | 4.13E-05 | 0.000219 |
| HAS2-AS1 | 1.1433535 | 0.5256202 | -1.12118 | 7.58E-10 | 1.65E-08 |
| LINC01082 | 1.632278 | 0.7507858 | -1.12041 | 1.24E-07 | 1.48E-06 |
| AC114488.1 | 5.8706723 | 2.7109547 | -1.11472 | 6.61E-12 | 3.19E-10 |
| AC026801.2 | 0.7125995 | 0.3324079 | -1.10014 | 4.75E-11 | 1.48E-09 |
| AL390198.1 | 3.1863688 | 1.491305 | -1.09534 | 2.19E-06 | 1.69E-05 |
| LINC01558 | 1.8303693 | 0.8625134 | -1.08552 | 8.35E-08 | 1.04E-06 |
| LINC01807 | 0.6969885 | 0.3317339 | -1.07111 | 0.000662 | 0.002313 |
| AC020659.1 | 0.7995922 | 0.3808242 | -1.07014 | 5.26E-05 | 0.000269 |
| AC009237.15 | 0.6669004 | 0.3178861 | -1.06896 | 3.19E-07 | 3.23E-06 |
| RHPN1-AS1 | 1.0821035 | 0.5163951 | -1.06729 | 8.35E-12 | 3.83E-10 |
| CASC19 | 5.5406001 | 2.6528251 | -1.06251 | 0.000335 | 0.001288 |
| PLBD1-AS1 | 2.4950602 | 1.2000162 | -1.05602 | 1.05E-11 | 4.68E-10 |
| AL139384.1 | 0.7480681 | 0.3598638 | -1.05572 | 7.39E-09 | 1.31E-07 |
| AL391056.1 | 1.4796837 | 0.715993 | -1.04727 | 4.10E-09 | 7.92E-08 |
| AL035661.1 | 15.400729 | 7.4584214 | -1.04606 | 1.90E-11 | 7.03E-10 |
| DRAIC | 0.6928008 | 0.3379828 | -1.03549 | 2.58E-05 | 0.000147 |
| AL163953.1 | 1.330536 | 0.6499695 | -1.03356 | 6.23E-08 | 8.09E-07 |
| AC004130.2 | 4.0784767 | 1.999985 | -1.02804 | 1.18E-12 | 6.41E-11 |
| LINC01315 | 5.0202642 | 2.4635063 | -1.02705 | 4.88E-10 | 1.09E-08 |
| AC104534.1 | 3.4407135 | 1.6891249 | -1.02643 | 0.001698 | 0.005124 |
| AL357079.1 | 1.1920022 | 0.5865443 | -1.02307 | 2.12E-13 | 1.60E-11 |
| AC004233.2 | 3.9543832 | 1.9473011 | -1.02198 | 4.44E-08 | 6.28E-07 |
| LINC00543 | 3.5753694 | 1.7686067 | -1.01548 | 5.20E-13 | 3.24E-11 |
| AC080129.2 | 1.3339045 | 0.6624584 | -1.00975 | 1.58E-10 | 4.17E-09 |
| AL031275.1 | 0.6378492 | 0.3168521 | -1.00941 | 0.00452 | 0.011693 |
| TDRKH-AS1 | 0.7588168 | 0.3771775 | -1.00851 | 2.04E-15 | 3.82E-13 |
| AC104958.2 | 5.1875345 | 2.578618 | -1.00845 | 4.35E-10 | 1.01E-08 |
| AL133520.1 | 1.8318814 | 0.9110602 | -1.00771 | 3.92E-11 | 1.26E-09 |
| MIR223HG | 0.4692352 | 0.9418152 | 1.005133 | 0.001573 | 0.004797 |
| AC005632.3 | 0.3537224 | 0.7153167 | 1.015965 | 8.84E-06 | 5.88E-05 |
| AC089999.4 | 0.3749677 | 0.7623585 | 1.023703 | 4.64E-05 | 0.000242 |
| AC129492.1 | 0.378213 | 0.7795386 | 1.043422 | 0.000234 | 0.000958 |
| AC115522.1 | 0.464065 | 0.9619892 | 1.051694 | 2.44E-07 | 2.59E-06 |
| AC130456.3 | 0.7906015 | 1.6469903 | 1.058809 | 0.010796 | 0.024158 |
| LINC02100 | 0.3780422 | 0.7876482 | 1.059004 | 0.022021 | 0.044037 |
| HAR1B | 0.437955 | 0.9135138 | 1.060644 | 1.91E-06 | 1.49E-05 |
| LOXL1-AS1 | 0.5591274 | 1.2157232 | 1.120566 | 2.59E-10 | 6.44E-09 |
| LINC01094 | 0.3753463 | 0.8202968 | 1.127924 | 1.90E-08 | 3.07E-07 |
| LINC01871 | 1.8826659 | 4.1434513 | 1.138056 | 1.83E-08 | 2.98E-07 |
| AC012317.1 | 0.5808119 | 1.2809706 | 1.141095 | 0.003335 | 0.00903 |
| AL132989.2 | 0.3706064 | 0.8208608 | 1.14725 | 3.09E-05 | 0.000172 |
| AC018410.1 | 0.307077 | 0.6832588 | 1.153832 | 0.022749 | 0.045161 |
| LINC02605 | 0.2501254 | 0.5627905 | 1.169946 | 2.31E-06 | 1.78E-05 |
| DLG3-AS1 | 0.470077 | 1.0614893 | 1.175121 | 0.013602 | 0.029056 |
| AC026202.2 | 0.2876774 | 0.6497719 | 1.175482 | 0.002445 | 0.006978 |
| AC069549.1 | 0.3476932 | 0.7918714 | 1.187451 | 0.01212 | 0.026476 |
| AC009163.6 | 0.3064565 | 0.7004525 | 1.192605 | 0.001587 | 0.00483 |
| AGAP1-IT1 | 0.8913642 | 2.0440187 | 1.197321 | 5.16E-07 | 4.83E-06 |
| AC090559.1 | 0.4569003 | 1.0585048 | 1.212076 | 0.008209 | 0.019159 |
| BX640514.2 | 0.2431068 | 0.5636329 | 1.213165 | 7.16E-07 | 6.42E-06 |
| AL136115.2 | 0.6381005 | 1.4797001 | 1.213449 | 0.012686 | 0.027505 |
| AP003555.1 | 0.353505 | 0.8337524 | 1.237888 | 0.00424 | 0.011 |
| LINC02688 | 0.382389 | 0.9149645 | 1.258675 | 3.63E-06 | 2.66E-05 |
| AC007996.1 | 0.5743775 | 1.3778396 | 1.262337 | 1.03E-13 | 8.96E-12 |
| AL121761.1 | 0.7096901 | 1.7051501 | 1.264638 | 0.000318 | 0.001232 |
| ADAMTSL4-AS1 | 0.240245 | 0.58251 | 1.277777 | 0.00322 | 0.008801 |
| AL606834.1 | 0.5921294 | 1.4612747 | 1.303243 | 1.45E-07 | 1.67E-06 |
| HLA-DQB1-AS1 | 0.7357994 | 1.8161341 | 1.303486 | 8.20E-07 | 7.21E-06 |
| AC007991.2 | 0.8795706 | 2.1939096 | 1.318633 | 0.00017 | 0.000742 |
| AC090181.2 | 1.4343041 | 3.5826005 | 1.320656 | 3.78E-08 | 5.40E-07 |
| AC005911.1 | 0.4033202 | 1.0077789 | 1.321182 | 3.22E-11 | 1.08E-09 |
| AC023090.1 | 0.2252044 | 0.5679969 | 1.334648 | 2.41E-07 | 2.57E-06 |
| AC025423.1 | 0.2366426 | 0.6157509 | 1.379637 | 0.006274 | 0.01534 |
| AC023825.2 | 0.244743 | 0.6372228 | 1.38053 | 0.001312 | 0.00413 |
| AC005392.2 | 1.911206 | 4.9929336 | 1.385404 | 5.71E-05 | 0.000288 |
| MIAT | 0.4635648 | 1.2217355 | 1.398089 | 2.78E-06 | 2.11E-05 |
| MAP3K5-AS1 | 0.3936624 | 1.0421815 | 1.404576 | 0.002474 | 0.007037 |
| LINC01443 | 0.2441738 | 0.6574074 | 1.428879 | 0.000652 | 0.002285 |
| AP005899.1 | 0.4739216 | 1.2892875 | 1.443854 | 1.75E-06 | 1.39E-05 |
| AP000753.2 | 0.2093402 | 0.5942212 | 1.505151 | 0.000688 | 0.002394 |
| LINC00941 | 0.4358538 | 1.2540938 | 1.524729 | 4.09E-09 | 7.92E-08 |
| AC005256.1 | 0.5384619 | 1.5556876 | 1.530636 | 3.50E-06 | 2.57E-05 |
| LINC02489 | 0.541774 | 1.6058798 | 1.567601 | 2.87E-07 | 2.94E-06 |
| TFAP2A-AS1 | 0.3796121 | 1.1451575 | 1.592948 | 3.56E-14 | 3.44E-12 |
| XXYLT1-AS2 | 0.2015604 | 0.652268 | 1.694253 | 6.14E-09 | 1.13E-07 |
| AL022316.1 | 0.5730463 | 1.8796992 | 1.713778 | 1.32E-07 | 1.55E-06 |
| AC064801.1 | 0.1833698 | 0.6126848 | 1.740389 | 1.80E-08 | 2.96E-07 |
| LUCAT1 | 0.192818 | 0.6443331 | 1.740567 | 0.017242 | 0.035677 |
| AL139022.1 | 0.1834427 | 0.6242961 | 1.766901 | 0.008125 | 0.019037 |
| AL157871.2 | 0.3009333 | 1.0434628 | 1.793864 | 3.94E-07 | 3.90E-06 |
| HIF1A-AS3 | 0.7838963 | 2.7522405 | 1.811872 | 0.00633 | 0.015436 |
| AC114760.2 | 0.2475614 | 0.8700847 | 1.813369 | 0.00028 | 0.001122 |
| LINC02195 | 0.3140163 | 1.165435 | 1.891957 | 5.40E-14 | 4.95E-12 |
| AC245128.3 | 0.2259796 | 0.8932124 | 1.982811 | 0.000215 | 0.000893 |
| UNC5B-AS1 | 0.3868872 | 1.59375 | 2.04244 | 1.53E-06 | 1.25E-05 |
| AL138789.1 | 0.1866623 | 0.7737306 | 2.051401 | 0.004081 | 0.010636 |
| AC010998.3 | 0.2417992 | 1.0023439 | 2.051496 | 0.000604 | 0.002145 |
| AC022784.1 | 0.2016332 | 0.8560651 | 2.085987 | 2.67E-10 | 6.54E-09 |
| AFAP1-AS1 | 1.0294031 | 4.896589 | 2.249969 | 0.003667 | 0.009763 |
| AC036176.3 | 0.1428079 | 0.8005559 | 2.486926 | 4.22E-05 | 0.000223 |
| AC092112.1 | 0.1411125 | 1.0003669 | 2.825611 | 1.27E-05 | 7.92E-05 |
| LINC02446 | 0.1928466 | 1.4061984 | 2.866275 | 1.91E-16 | 5.55E-14 |
| DLGAP1-AS5 | 0.0277386 | 1.4504399 | 5.708452 | 7.38E-07 | 6.59E-06 |

*GU, genome unstable; GS, genome stable; FC, fold change.*
