# Supplementary material for: Identification of a Genome Instability-Associated LncRNA Signature for Prognosis Prediction in Colon Cancer
Source: Front Genet. 2021 Jun 7;12:679150. doi: 10.3389/fgene.2021.679150 (PMC8215581; doi:10.3389/fgene.2021.679150)
Supplement: Supplementary file 3 [file Table_2.DOCX]

Supplementary Material

**TABLE S2 |** Baseline clinical characteristics of the training set, testing set and TCGA set

| **Covariates** | **Type** | **TCGA set (n/%)** | **Testing set (n/%)** | **Training set (n/%)** | ***P* value** |
| --- | --- | --- | --- | --- | --- |
| Age | <=65 | 175(41.87%) | 91(43.75%) | 84(40%) | 0.498 |
|  | >65 | 243(58.13%) | 117(56.25%) | 126(60%) |  |
| Gender | Female | 193(46.17%) | 90(43.27%) | 103(49.05%) | 0.277 |
|  | Male | 225(53.83%) | 118(56.73%) | 107(50.95%) |  |
| Stage | Stage I-II | 233(55.74%) | 105(50.48%) | 128(60.95%) | 0.055 |
|  | Stage III-IV | 174(41.63%) | 96(46.15%) | 78(37.14%) |  |
|  | unknow | 11(2.63%) | 7(3.37%) | 4(1.9%) |  |
| T stage | T1-2 | 84(20.1%) | 42(20.19%) | 42(20%) | 1.000 |
|  | T3-4 | 334(79.9%) | 166(79.81%) | 168(80%) |  |
| M stage | M0 | 312(74.64%) | 154(74.04%) | 158(75.24%) | 0.425 |
|  | M1 | 57(13.64%) | 32(15.38%) | 25(11.9%) |  |
|  | unknow | 49(11.72%) | 22(10.58%) | 27(12.86%) |  |
| N stage | N0 | 248(59.33%) | 114(54.81%) | 134(63.81%) | 0.076 |
|  | N1-2 | 170(40.67%) | 94(45.19%) | 76(36.19%) |  |
